# MOFs-Derived Three-Phase Microspheres: Morphology Preservation and Electromagnetic Wave Absorption

**DOI:** 10.3390/molecules27154773

**Published:** 2022-07-26

**Authors:** Xin Yang, Tie Shu, Xianfeng Yang, Min Qiao, Dashuang Wang, Xinghua Li, Jinsong Rao, Zhaohui Liu, Yuxin Zhang, Pingan Yang, Kexin Yao

**Affiliations:** 1 Multi-Scale Porous Materials Center, Institute of Advanced Interdisciplinary Studies & School of Chemistry and Chemical Engineering, Chongqing University, Chongqing 400044, China; xyang0610@163.com (X.Y.); shutie950112@163.com (T.S.); 202018021088@cqu.edu.cn (M.Q.); zhaohui.liu@cqu.edu.cn (Z.L.); 2 State Key Laboratory of Photon-Technology in Western China Energy, School of Physics, Northwest University, Xi’an 710127, China; 202020719@stumail.nwu.edu.cn (X.Y.); xinghua.li@nwu.edu.cn (X.L.); 3 College of Material Science and Engineering, Chongqing University, Chongqing 400044, China; 20210901021@cqu.edu.cn (D.W.); zhangyuxin@cqu.edu.cn (Y.Z.); 4 School of Automation, Chongqing University of Posts and Telecommunications, Chongqing 400065, China; yangpa@cqupt.edu.cn

**Keywords:** electromagnetic wave absorption, MOFs, nanoparticles, metal oxide

## Abstract

Reasonable structural design and composition control are the dominant factors for tuning the electromagnetic absorbing properties of materials. In this paper, microspheres composed of NiO, Ni, and Co_3_O_4_ nanoparticles (NCMO) were successfully synthesized using a mild oxidation method. Benefiting from the multi-component composition and a unique microstructure, the RL_min_ of CNMO can reach −46.8 dB at 17 GHz, with an effective absorption bandwidth of 4.1 GHz (13.9–18 GHz). The absorbing properties and the absorbing mechanism analysis showed that the microsphere-structured NCMO composed of multi-component nanoparticles enhanced the interface polarization, thereby improving the absorption performance. This research provides a new avenue for MOF-derived oxide materials with excellent electromagnetic wave absorbing properties.

## 1. Introduction

With the development of electronic technology, various electronic devices which bring great convenience to human life have been continuously updated. However, this development is accompanied by electromagnetic radiation pollution, which endangers human health, interferes with communication, and undermines industrial safety [1,2,3,4]. An efficient path to solve electromagnetic pollution is to apply electromagnetic wave (EMW) absorbing materials to block/absorb EMW. In order to adapt to various application environments, EMW absorbing materials should possess the characteristics of light weight, thin thickness, a wide absorption frequency band, good absorption capacity, and high thermal stability [5,6].

Among the traditional absorbing materials, magnetic metals have been widely studied due to their low cost and excellent magnetic loss performance [7]. It is worth noting that Ni- and Co-based oxides have dual decay mechanisms of magnetic loss and dielectric loss [8,9,10]. It is generally known that the evaluation of EMW absorption characteristics is composed of complex permittivity, complex permeability, and impedance matching, which are closely related to the composition and morphology of EMW absorbing materials. However, the absorption performance of a single magnetic material is not so satisfactory due to poor impedance matching. To address this issue, the integration of multi-component magnetic particle materials can build more interfaces to adjust the impedance matching and improve the attenuation capacity [11]. Therefore, rational structural design and composition selection are effective strategies to enhance the absorption performance. In recent years, multi-component Ni- and Co-based oxides with different morphologies have been successfully synthesized and applied in EMW absorption. For example, the core-shell structured Co_3_O_4_@NiCo_2_O_4_ [12], Co NPs/porous carbon spheres [13], etc., provide inspiration for the structural design of metal oxides.

Metal-organic frameworks (MOFs) have become popular template precursors for material synthesis due to their tunable pore structure and microstructure, and are widely applied in the field of gas adsorption, energy storage, and catalysis [14]. Similarly, MOFs also show great potential in the field of electromagnetic wave absorption. For instance, Qiu et al. [15] prepared N-doped carbon-coated Ni microspheres exhibiting an effective absorption bandwidth of 11.2 GHz and a reflection loss (RL) of −55.1 dB at 1.8 mm. Cui et al. fabricated nanocages with Ni doping by vacuum carbonization, and achieved an effective absorption bandwidth of 12.6 GHz when the absorber thickness varied from 1–5 mm [16]. However, the obtained products are mostly pure metals or a small number of metal oxides (the oxygen element originates from the organic ligands of MOFs) through the previous strategies of calcining MOFs under vacuum or inert gas. Calcined in an inert gas, the generating amorphous carbon can form a conductive network. More importantly, the depletion of C, N, and O during calcination leads to the collapse of the MOFs’ structure [17]. Multicomponent magnetic materials have been proven to show stronger dielectric loss properties compared to single metal compositions [18]. Besides composition, careful design of nanostructures is also critical for EMW absorption performance [19,20]. Therefore, the best way to prepare MOF derivatives by calcination in air and to make the products fully inherit the structure of MOFs precursors is still a challenge.

Accordingly, in this work, we propose a reasonable approach to prepare microspheres composed of NiO, Ni, and Co_3_O_4_ nanoparticles (NCMO) through mild oxidation of Ni/Co-MOFs. During the mild oxidation process, NCMO inherits the microsphere structure of Ni/Co MOFs. The Ni cations in MOFs is partially oxidized to NiO, the rest is converted to Ni metal, and the Co cations are completely converted to Co_3_O_4_. These transformed species agglomerate into particles with diameters of about 10–20 nm. As a result, NCMO own abundant interfaces, which are desired and favorable for interfacial polarization. The multicomponent microsphere structure composed of NiO, Ni, and Co_3_O_4_ enables multiple scattering and dipole polarization. The results provide inspiration for the application of MOF derivatives in the field of electromagnetic wave absorption.

## 2. Materials and Methods

### 2.1. Materials

The materials required, namely Ni(NO_3_)_2_·6H_2_O, Co(NO_3_)_2_·6H_2_O, 1,3,5-Benzenetricarboxylic acid (H_3_BTC), and N, N-Dimethylformamide (DMF) were all obtained from Shanghai Aladdin Biochemical Technology Co., Ltd. (Shanghai, China). All reagents were of analytical grade and used without any further purification.

### 2.2. Synthesis of Ni/Co-MOFs and Ni-MOFs

The synthesis of Ni/Co-MOF follows the typical method with some modifications. Here, 0.873 g of Ni(NO_3_)_2_·6H_2_O, 0.291 g of Co(NO_3_)_2_·6H_2_O, and 0.219 g of H_3_BTC were magnetically stirred in 30 mL of DMF for 30 min to obtain a homogeneous solution. The solution was then transferred to a Teflon-lined stainless steel autoclave and kept at 120 °C for 18 h. Subsequently, the resulting precipitate was collected by centrifugation, washed with DMF for several times, and dried at 80 °C for 12 h t to obtain Ni/Co-MOF. As a control sample, the preparation process and reagent dosage of Ni-MOF were similar to the above method, although no Co(NO_3_)_2_·6H_2_O was added.

### 2.3. Preparation of NMO and NCMO

A fast heating rate would lead to the structural collapse of the MOF. To solve this problem, Ni-MOF and Ni/Co-MOF were put into a tubular furnace, raised to 400 °C with a slow heating rate (1 °C min^−1^) and kept for 40 min in air. The Ni-MOF and Ni/Co-MOF calcined powder were collected, and then marked as NMO and NCMO, respectively.

### 2.4. Characterization

The microscopic morphology and particle size of the samples were characterized using scanning electron microscopy (SEM, Helios5,Thermo Scientific, Waltham, MA, USA) and transmission electron microscopy (TEM, Talos, F200S, Thermo Scientific, Waltham, MA, USA). The crystal structure and phase composition were analyzed using X-ray diffraction (XRD, Empyrean, Panalytical B.V., Netherlands) with Cu Kα radiation between 5° and 85° (40 kV; 40 mA; 5° min^−1^). Additionally, X-ray photoelectron spectroscopy (XPS, K-Alpha, Thermo Scientific, Waltham, MA, USA) was obtained using a Thermo Scientific Kα energy spectrometer paired with an X-ray source of monochromatic Al-Kα. The NMO (50 wt%) or NCMO (50 wt%) were uniformly mixed with paraffin wax (50 wt%) and pressed into a concentric ring with an outer diameter of 7.0 mm, an inner diameter of 3.04 mm, and a thickness of 3.04 mm. The electromagnetism parameters of NMO and NCMO, in the frequency range of 2–18 GHz, were obtained using a vector network analyzer (Agilent N5234A, Santa Clara, CA, USA) using the coaxial method.

## 3. Results Discussion

**Figure 1:**
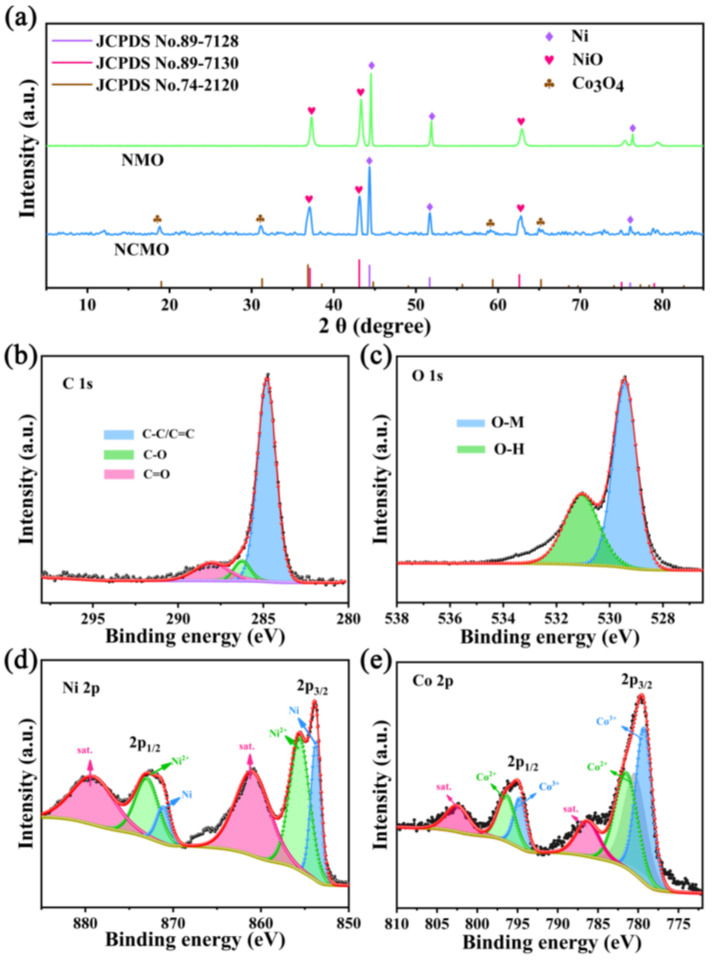
(**a**) XRD patterns of NCMO and NMO; XPS spectra of NCMO: (**b**) C 1s; (**c**) O 1s; (**d**) Ni 2p; (**e**) Co 2p.

The X-ray diffraction (XRD) patterns of Ni-MOF and Ni/Co-MOF are shown in Figure S1. In addition, Co-MOFs were synthesized by the same method, and XRD diffraction proved that Co can act as the central element to link organic ligands to form MOFs (Figure S1). The diffraction peaks of the XRD pattern are the same as in previous reports (CCDC No. 1274034) [21,22]. For Ni/Co-MOF, the XRD diffraction peaks are similar to those of Ni-MOF due to the close atomic radius of Ni and Co. Combining the corresponding elemental mapping (Figure S2) of Ni-MOF and Ni/Co-MOF confirms that they were successfully synthesized. Figure 1a shows the XRD patterns of NMO and NCMO. For the NMO, the diffraction peaks at 37.1°, 43.1°, and 62.6° belong to the (111), (200), and (220) crystalline planes of NiO, respectively. Furthermore, there are three diffraction peaks at 44.3°, 51.7°, and 76.1°, indicating the presence of elemental Ni. For NCMO, due to the introduction of Co, the diffraction peaks at 19.0°, 31.3°, 59.3° and 65.2° in the diffraction pattern of NCMO belong to the (111), (222), (511), and (440) crystalline planes of Co_3_O_4_, respectively. In addition, XPS (X-ray photoelectron spectroscopy) was used to characterize the surface chemical composition and chemical bonding state of the as-synthesized materials. As shown in Figure S3, the wide-scan XPS spectrum exhibits the existence of C, O, Ni, and Co elements. The high-resolution XPS spectrum of C 1s (Figure 1b) can be deconvoluted into two peaks, which are ascribed to graphitized C-C or C=C (284.8 eV) and C-O (281.5 eV) [23,24]. For the high-resolution XPS spectrum of O 1s (Figure 1c), the peaks at 529.4 and 531.1 eV are assigned to O-M (Co-O or Ni-O) and –OH species (absorbed on the surface of as-prepared composite), respectively [25]. In the spectrum of Ni 2p (Figure 1d), the peaks at 853.7 and 871.1 eV are ascribed to Ni 2p_3/2_ and Ni 2p_1/2_, and the peaks at 855.5 and 872.9 eV are assigned to divalent (Ni^2+^ 2p_3/2_ and Ni^2+^ 2p_1/2_) oxidative states of Ni. Moreover, the other two peaks located at 860.9 and 879 eV are assigned to shake-up satellite peaks (denoted as “Sat.”) [26,27]. The spectrum of Co 2p is shown in Figure 1e, in which two large peaks at 779.8 and 795.1 eV are ascribed to Co 2p_3/2_ and Co 2p_1/2_. Furthermore, both peaks of Co 2p_3/2_ and Co 2p_1/2_ can be deconvoluted into four peaks, corresponding to the Co^3+^ and Co^2+^, respectively [28]. Meanwhile, the peaks at 786.2 and 802.4 eV are satellite peaks belonging to Co 2p_3/2_ and Co 2p_1/2_, respectively [29]. Correspondingly, the XPS analysis of NMO is placed in the Supplementary Materials (Figure S4).

**Figure 2:**
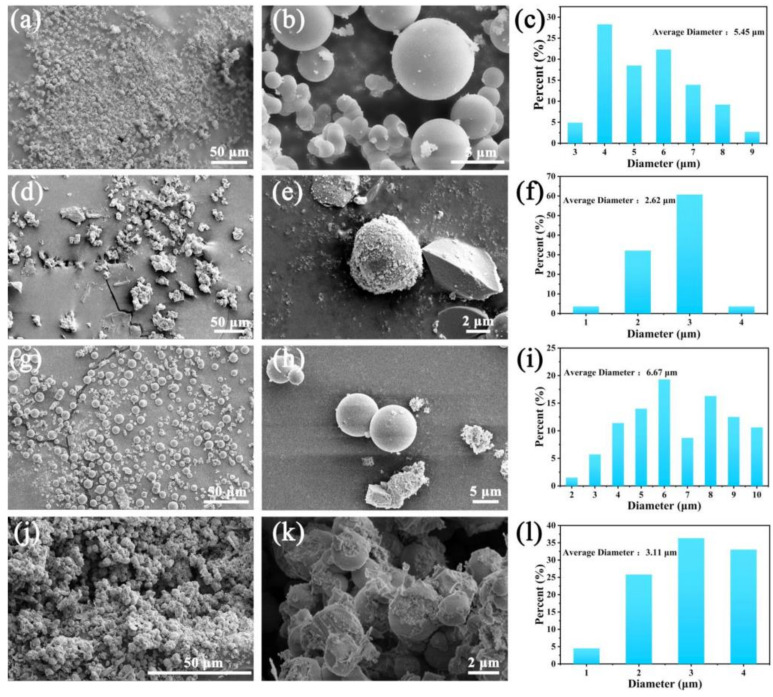
SEM images of (**a**,**b**) Ni-MOF; (**d**,**e**) NMO; (**g**,**h**) Ni/Co-MOF; (**j**,**k**) NCMO. Particle size of (**c**) Ni-MOF; (**f**) NMO; (**i**) Ni/Co-MOF; (**l**) NCMO.

Additionally, Ni-MOF was further characterized by SEM (scanning electron microscopy), as shown in Figure 2a,b. The microscopic morphology of Ni-MOF is spherical with regular surface. We made statistics on the size of the Ni-MOF spheres, as shown in Figure 2c, and determined that the average diameter of Ni-MOF spheres is 5.45 μm. After thermal treatment in air, Ni cations in Ni-MOF were in-situ converted to NiO and Ni, and the overall morphology remained spherical. The difference is that the surface of NMO is decorated with granular substances (Figure 2d,e). The average particle size of NCMO is 2.62 μm (Figure 2f), which is smaller than that of Ni-MOF. This is because the calcination of Ni-MOFs in air inevitably leads to the consumption of C and N, resulting in the shrinkage of spherical MOFs. In addition, the ramping rate has a huge influence on the structure when the MOFs are thermally treated [17]. Figure S5 shows the SEM image of Ni-MOF and Ni/Co-MOF after calcination at a ramping rate of 3 °C min^−1^. The spherical structure completely collapsed due to the greater heating rate. Therefore, the lower heating rate was conducted (1 °C min^−1^) to treat the MOFs, which largely preserved the structure of the spherical precursors. Figure 2g,h show the SEM images of Ni/Co-MOF under different magnifications. The morphology of Ni/Co-MOF is also spherical. Due to the introduction of Co, the size (6.67 μm) of Ni/Co-MOF is larger than Ni-MOF, as shown Figure 2i. After calcination, the size of NCMO was reduced to 3.11 μm (Figure 2l). Likewise, the surface of NCMO is covered with small particles (Figure 2j,k). It can be concluded from the above analysis that the spherical composite material composed of NiO, Ni, and Co_3_O_4_ was successfully obtained by the slow heating treatment.

**Figure 3:**
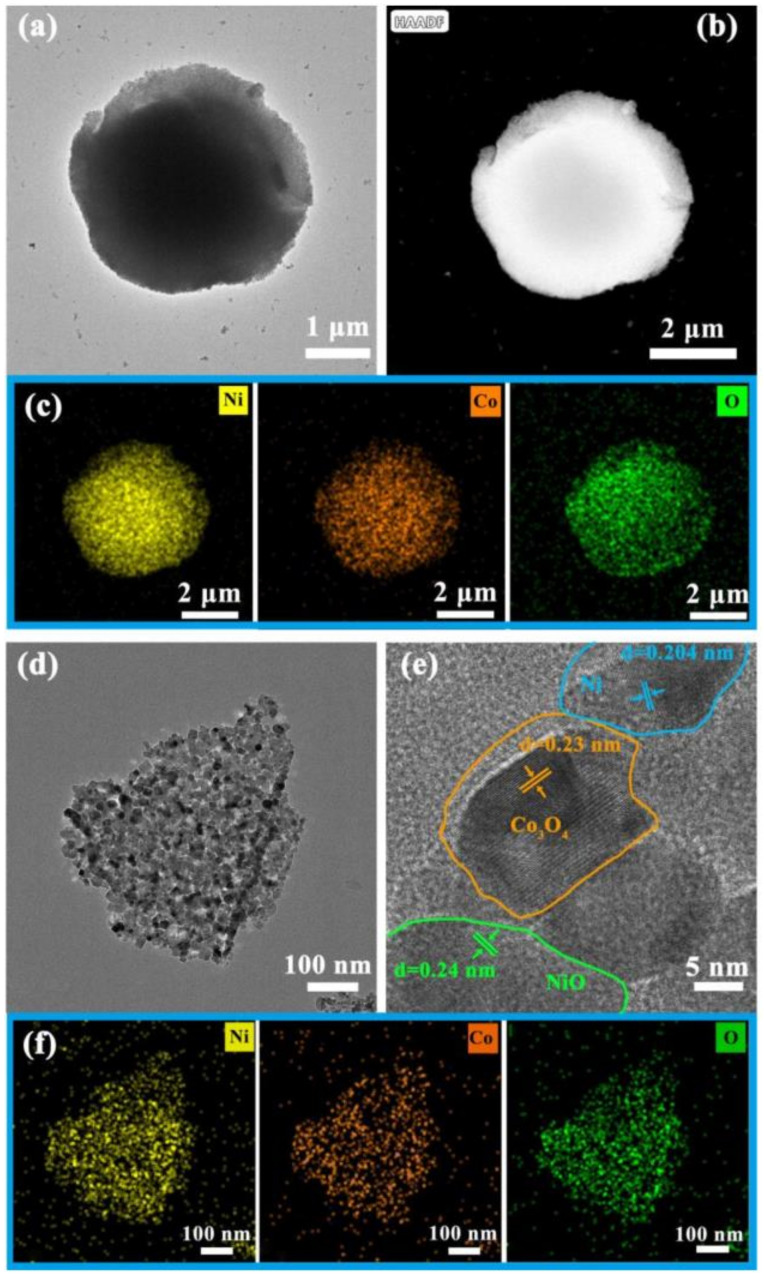
(**a**) TEM image of NCMO; (**b**) HAADF-STEM image of NCMO; (**c**) Corresponding elemental mapping; (**d**) TEM image of debris of NCMO; (**e**) HRTEM images of debris of NCMO; (**f**) Corresponding elemental mapping.

The as-prepared NMO and NCMO were investigated using TEM (transmission electron microscopy) in order to further reveal their microstructure. From the TEM and HAADF-STEM images of NCMO (Figure 3a,b), it is obvious that the NCMO sphere is composed of a large number of tiny particles. The corresponding elemental mapping indicates the existence of Ni, Co and O (Figure 3c). There are some debris scattered around the sphere, which possibly detached from the sphere during the ultrasonic preparation of the TEM sample. It is worth noting that the debris are composed of a large number of nanoparticles with a size of approximately 10–20 nm (Figure 3d). This structure might originate from the agglomeration of metals in Ni/Co-MOF during calcination. The high-resolution TEM (HRTEM) image of the particles is shown in Figure 3e. The lattice fringes of 0.23 nm, 0.24 nm, and 0.204 nm are matched with the (222) lattice planes of Co_3_O_4_, (006) lattice planes of NiO, and (111) lattice planes of Ni, respectively. The corresponding elemental mapping results are also exhibited in Figure 3f. Similarly, the uniform distribution of Ni, Co and O elements can be observed. In light of the above, NCMO has a microsphere structure, composed of NiO, Ni, and Co_3_O_4_ nanoparticles in the range of 10–20 nm. The heat treatment at a low heating rate can agglomerate the metal elements in the Ni/Co-MOF to form nanoparticles, while maintaining the microsphere structure. The TEM and HAADF-STEM images of NMO, and the corresponding elemental mapping result shown in Figure S6a,b indicates the uniform distribution of Ni and O elements (Figure S6c–e).

The electromagnetic wave absorbing performance of NMO and NCMO is evaluated by reflection loss (RL), which is calculated using the following formula [30,31]:

(1) $$\begin{array}{c} Z=\left|\frac{Z_{in}}{Z_{0}}\right|=\sqrt{\left|\mu_{r}/\varepsilon_{r}\right|}\tan h\left[j\left(\frac{2\pi fd}{c}\right)\sqrt{\mu_{r}\varepsilon_{r}}\right] \end{array}$$

(2) $$R_{L}\left(\mathrm{dB}\right)=20{log}_{10}\left|\frac{Z_{in}-Z_{0}}{Z_{in}+Z_{0}}\right|$$

where $Z_{in}$ is the input impedance of the absorber; $Z_{0}$ is the impedance of free space; $\varepsilon_{r}=\varepsilon^{\prime }-j\varepsilon^{\prime \prime }$ represents the relative complex permittivity; $\mu_{r}=\mu^{\prime }-j\mu^{\prime \prime }$ represents the complex permeability; *f* is the frequency of the microwave; *d* is the thickness of absorber and *c* is the speed of light.

**Figure 4:**
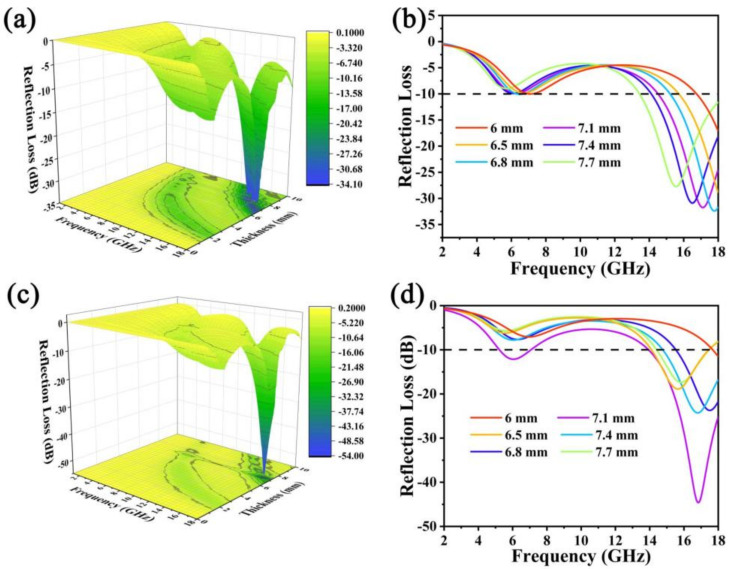
The 3D reflection loss map of (**a**) NMO; (**c**) NCMO. Reflection loss curves of (**b**) CNMO; (**d**) NMO.

The 3D projection diagrams (Figure 4a,c) exhibit the variation of RL with increasing microwave frequency. When the RL value is below −10 dB, 90% of the incident electromagnetic wave is completely absorbed, and the corresponding frequency width is called the effective absorption bandwidth (EAB) [32]. Following this criterion, the absorbing properties of NMO and NCMO are discussed. The 3D projection diagrams of NMO (Figure 4a) and NCMO (Figure 4c) exhibit the variation of RL with increasing microwave frequency. For NMO (Figure 4b), when the thickness is 7.1 mm, the RL_min_ is −33.48 dB with the EAB of 3.6 GHz (14.9–18 GHz). When the thickness is increased to 7.7 mm, the RL_min_ is −27.7 dB, and the EAB is 4.1 GHz (13.9–18 GHz). Figure 4d shows the relationship between frequency and reflection loss of NCMO. By contrast, when the thickness is 7.1 mm for NCMO, the RL_min_ reaches −46.8 dB at 17 GHz, and the EAB is 4.1 GHz (13.9–18 GHz). This indicates that the multi-component NCMO has better electromagnetic wave absorption properties, possibly due to the introduction of Co_3_O_4_ nanoparticles.

**Figure 5:**
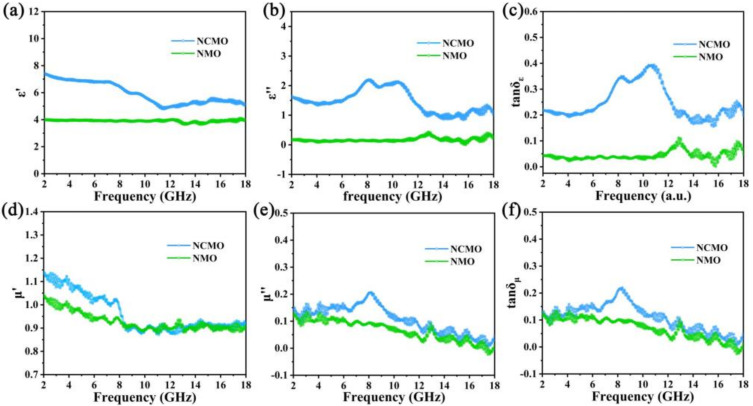
Frequency dependence of electromagnetic parameters of NMO and NCMO: (**a**) real part ($\varepsilon^{\prime }$); (**b**) imaginary part ($\varepsilon^{\prime \prime }$); (**c**) dielectric loss (tan$\delta_{\varepsilon }$); (**d**) real part ($\mu^{\prime }$); (**e**) imaginary part ($\mu^{\prime \prime }$); (**f**) magnetic loss (tan$\delta_{\mu }$).

Relative complex permittivity ($\varepsilon_{r}=\varepsilon^{\prime }-j\varepsilon^{\prime \prime }$) and relative complex permeability ($\mu_{r}=\mu^{\prime }-j\mu^{\prime \prime }$) are two important parameters to determine the microwave absorption performance. The real part ($\varepsilon^{\prime }$ and $\mu^{\prime }$) represents the ability to store electromagnetic wave energy. The imaginary part ($\varepsilon^{\prime \prime }$ and $\mu^{\prime \prime }$) is related to the dissipation of electromagnetic wave energy [33]. Figure 5a,b clearly show that NCMO has larger $\varepsilon^{\prime }$ and $\varepsilon^{\prime \prime }$, indicating stronger energy storage capacity and polarization loss. In addition, polarization peaks appear in the curve of $\varepsilon^{\prime \prime }$ versus frequency, indicating conductance loss and multiple polarization relaxation in the NCMO. Figure 5d,e are the relative complex permeability of NMO and NCMO, and it is obvious that the samples exhibit multi-resonance phenomena, which may be due to the anisotropic distribution of different nanoparticles. The main source of magnetic anisotropy in NCMO is the effective shape anisotropy of the nanostructures composed of these nanoparticles [34]. Additionally, in the range of 2–18 GHz, the complex permeability of NCMO is larger than that of NMO, indicating that the magnetic loss capability of NCMO is better than that of NMO. It is worth noting that tan$\delta_{\mu \;}$ is larger than tan$\delta_{\varepsilon }$ (Figure 5c,f), indicating that dielectric loss plays a dominant role in microwave loss.

**Figure 6:**
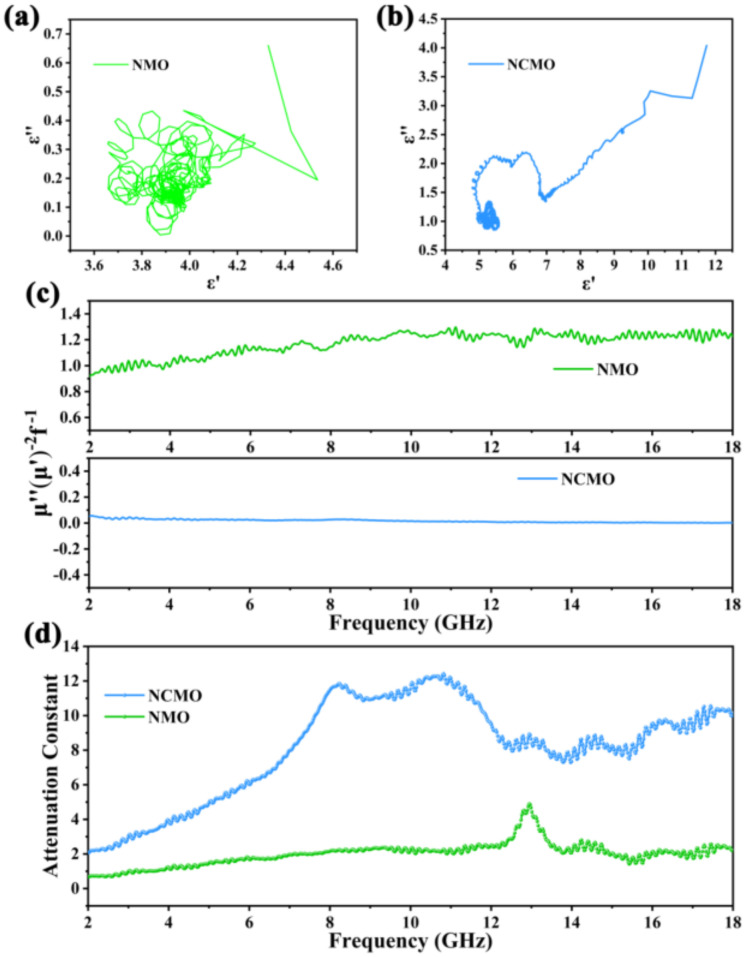
(**a**) Cole–Cole plots of NMO; (**b**) Cole–Cole plots of NCMO; (**c**) C0 values of NMO and NCMO; (**d**) Attenuation constants (α) of NMO and NCMO.

To further investigate the mechanism of dielectric loss, the Cole–Cole curves of NMO and CNMO are depicted (Figure 6a,b). These are based on the following formula [35,36]:

(3) $${\left(\varepsilon^{\prime }-\frac{\varepsilon_{s}+\varepsilon_{\infty }}{2}\right)}^{2}+={\left(\frac{\varepsilon_{s}-\varepsilon_{\infty }}{2}\right)}^{2}$$

where $\varepsilon_{s}$ and $\varepsilon_{\infty }$ refer to the static permittivity and relative dielectric permittivity at the high-frequency limit, respectively.

The semicircles in the curve are called Cole–Cole semicircles, and each semicircle represents a Debye relaxation process of the material. Both NMO and NCMO have multiple semicircles, indicating that they both have Debye relaxation processes. In addition, the Cole–Cole semicircle of NCMO and NMO is irregular, which means that in addition to Debye relaxation, other dielectric loss mechanisms, such as electron polarization and dipole polarization, should also be considered. The interface between NiO, Ni, and Co_3_O_4_ nanoparticles resulted in interface polarization due to charge aggregation. In general, magnetic losses include natural resonance, eddy current loss, exchange resonance, domain wall resonance, and hysteresis loss. When the value C_0_ ($C_{0}=\;u^{\prime \prime }{\left(u^{\prime }\right)}^{-2}f^{-1}$) does not vary with frequency, that is, the magnetic loss mainly originates from the eddy current loss [37]. As shown in Figure 6c, the C_0_ curve of NCMO is closer to a straight line, and the value of C_0_ does not change significantly with increasing frequency, indicating that eddy current loss is the dominant mechanism of magnetic loss [38]. In order to further evaluate the microwave absorption capacity of the material, the attenuation constant is obtained through the electromagnetic parameters, and the calculation equation is as follows [39]:

(4) $$\alpha =\frac{\sqrt{2}\pi f}{c}\sqrt{\mu^{\prime \prime }\;\varepsilon^{\prime \prime }-\;\mu^{\prime }\;\varepsilon^{\prime }+\sqrt{{\left(\;u^{\prime \prime }\;\varepsilon^{\prime \prime }-\;u^{\prime }\;\varepsilon^{\prime }\right)}^{2}+{\left(\;u^{\prime }\;\varepsilon^{\prime \prime }+\;u^{\prime \prime }\;\varepsilon^{\prime }\right)}^{2}}}$$

The larger the attenuation coefficient, the stronger the loss ability of the material to electromagnetic waves. Figure 6d shows the attenuation constant of NMO and NCMO. Obviously, the decay constant of NCMO is larger. In the high frequency range, the fluctuation of the attenuation constant of NCMO indicates that the existence of polarization relaxation has a good attenuation effect on electromagnetic waves of a specific frequency. Due to the microsphere structure formed by the close packing of three-phase nanoparticles, electromagnetic wave conduction needs to pass through more interfaces, resulting in excellent conduction loss of NCMO. The multiphase interface also enhances interfacial polarization and dipole polarization, which is responsible for the better absorption performance of NCMO.

## 4. Conclusions

Spherical absorbing materials composed of NiO, Ni, and Co_3_O_4_ nanoparticles were successfully synthesized by slow heating calcination. Compared with NMO, benefiting from the introduction of Co, more interfaces in NCMO are created, leading to superior conduction loss, stronger interface polarization, and dipole polarization. The as-synthesized NCMO displays a broad EAB of 4.1 GHz (13.9–18 GHz) at the corresponding RL_min_ value of −46.8 dB. The rational design and synthesis on structure and components is the premise to ensure the excellent performance of absorbing materials. The proposed strategy in this work may provide inspiration and possibilities for directly obtaining oxidized derivatives of MOFs for electromagnetic wave absorption.

## Data Availability

Data of the compounds are available from the authors. Informed consent was obtained from all subjects involved in the study.

## Supplementary Materials

The following supporting information are available online at https://www.mdpi.com/article/10.3390/molecules27154773/s1, Figure S1: XRD patterns of Ni-MOF and Ni/Co-MOF; Figure S2: (a) TEM image of NMO; (b) HAADF-STEM image corresponding elemental mapping of NMO; (c) TEM image of NCMO; (d) HAADF-STEM image corresponding elemental mapping of NCMO; Figure S3: The wide-scan XPS spectrum of NCMO; Figure S4: XPS spectrum of NMO; Figure S5: (a,b) SEM images of NMO (3 °C min^−1^); (c,d) SEM images of NCMO (3 °C min^−1^); Figure S6: (a) SEM image of NMO; (b) HAADF-STEM image of NMO; (c–e) elemental mapping of NMO.

## Sample Availability

Samples of the compounds are available from the authors.
