# Supplementary material for: MOFs-Derived Three-Phase Microspheres: Morphology Preservation and Electromagnetic Wave Absorption
Source: Molecules. 2022 Jul 26;27(15):4773. doi: 10.3390/molecules27154773 (PMC9332212; doi:10.3390/molecules27154773)
Supplement: Supplementary file 1 [file molecules-27-04773-s001.zip › molecules-1800095-supplementary.pdf]

Supplementary Information

MOFs-Derived Three-Phase Microspheres: Morphology Preservation and Electromagnetic Wave Absorption

Xin Yang [1,†], Tie Shu [1,†], Xianfeng Yang [2], Min Qiao [1], Dashuang Wang [3], Xinghua Li [2], Jinsong Rao [3,*],
Zhaohui Liu [1], Yuxin Zhang [3], Pingan Yang [4] and Kexin Yao [1,*]

1 Multi-Scale Porous Materials Center, Institute of Advanced Interdisciplinary Studies, & School of Chemistry and Chemical Engineering, Chongqing University, Chongqing 400044, China; xyang0610@163.com (X.Y.); shutie950112@163.com (T.S.); 202018021088@cqu.edu.cn (M.Q.); zhaohui.liu@cqu.edu.cn (Z.L.)
2 State Key Laboratory of Photon-Technology in Western China Energy, School of Physics, Northwest
University, Xi'an 710127, China; 202020719@stumail.nwu.edu.cn (X.Y.); xinghua.li@nwu.edu.cn (X.L.)
3 College of Material Science and Engineering, Chongqing University, Chongqing 400044, China; 20210901021@cqu.edu.cn (D.W.); zhangyuxin@cqu.edu.cn (Y.Z.)
4 School of Automation, Chongqing University of Posts and Telecommunications, Chongqing 400065, China; yangpa@cqupt.edu.cn
* Correspondence: rjs@cqu.edu.cn (J.R.); kexinyao@cqu.edu.cn (K.X.Y.)
† These authors contributed equally to this work.

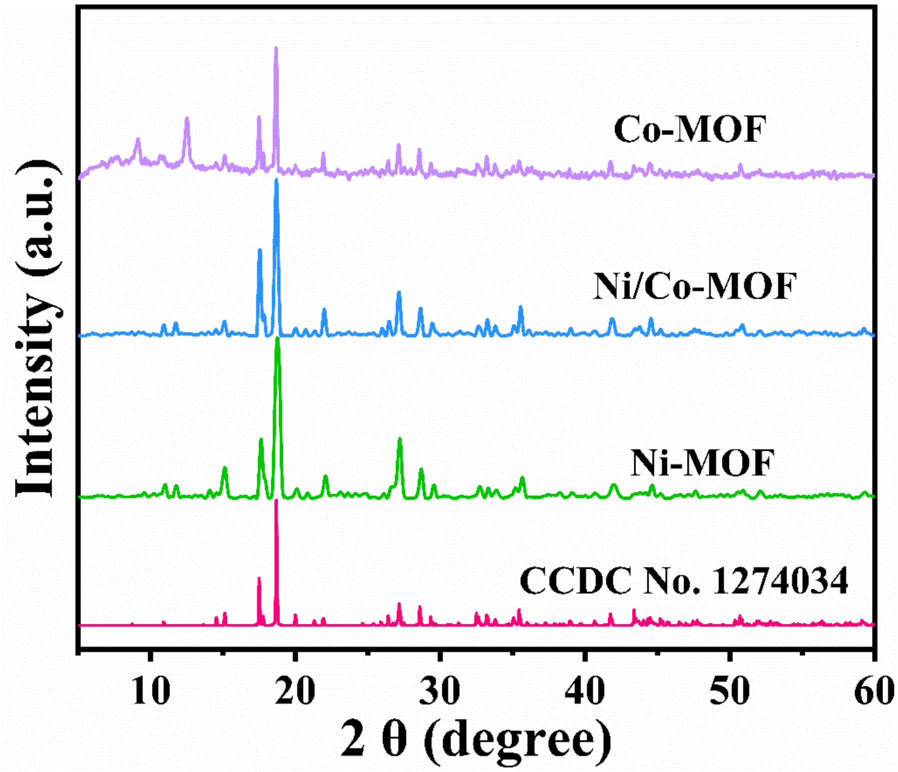

Figure S1. XRD patterns of Ni-MOF, Co-MOF and Ni/Co-MOF.

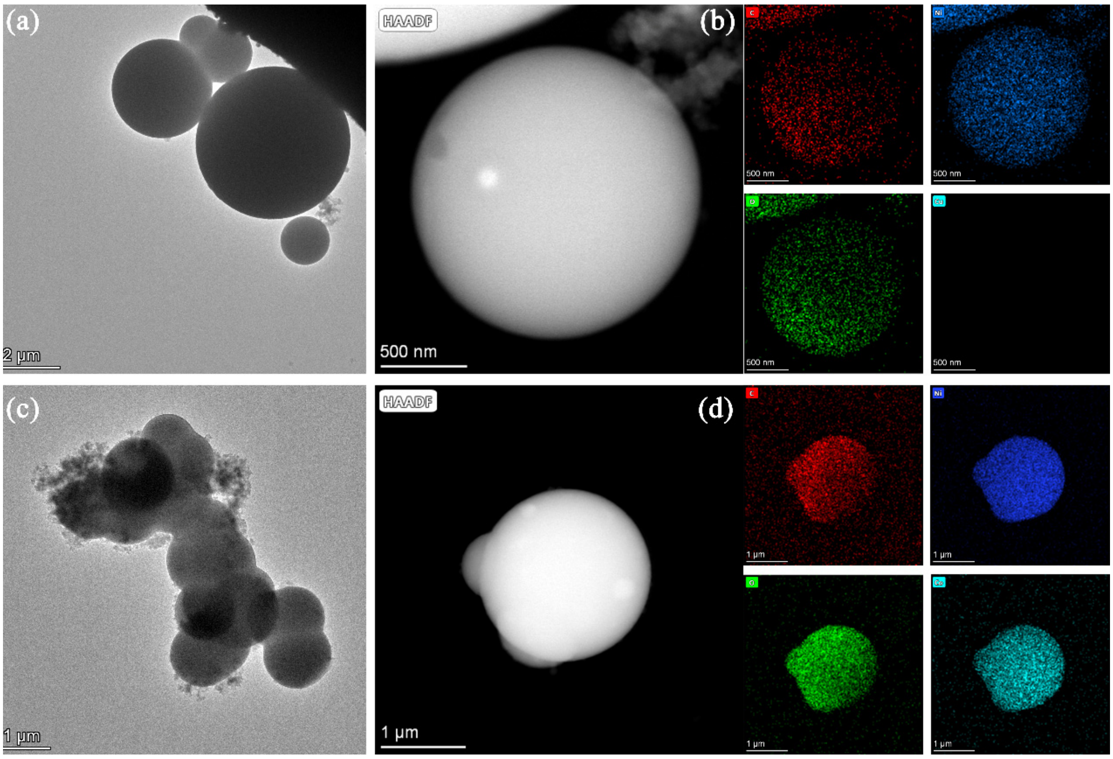

Figure S2. (a) TEM image of Ni-MOF; (b) HAADF-STEM image corresponding elemental mapping of Ni-MOF; (c) TEM image of Ni/Co-MOF; (d) HAADF-STEM image corresponding elemental mapping of Ni/Co-MOF

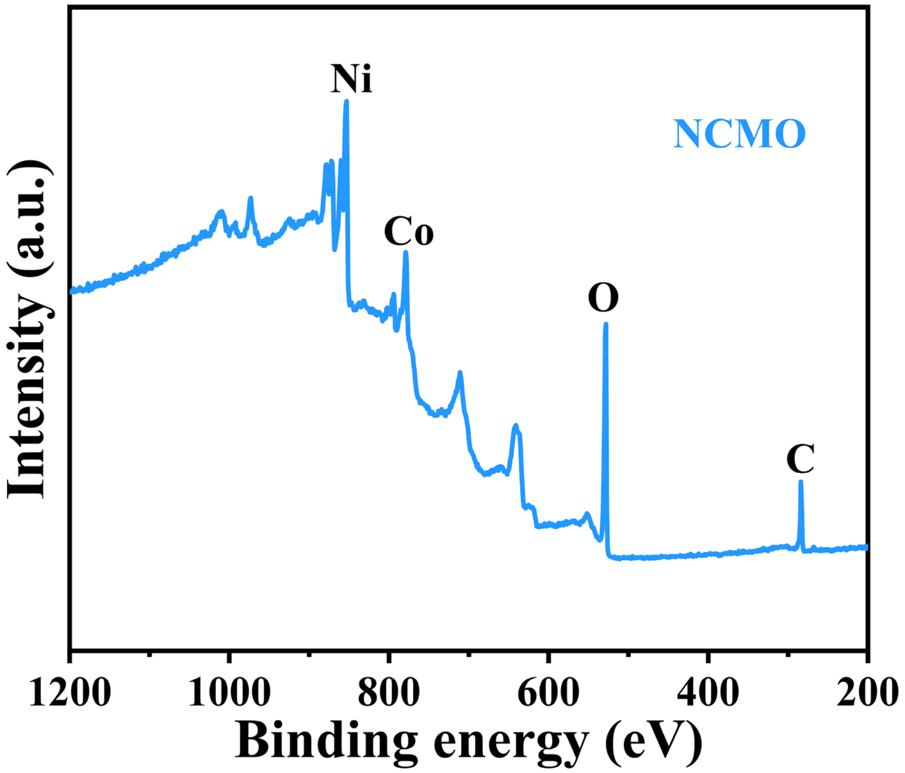

Figure S3. The wide-scan XPS spectrum of NCMO

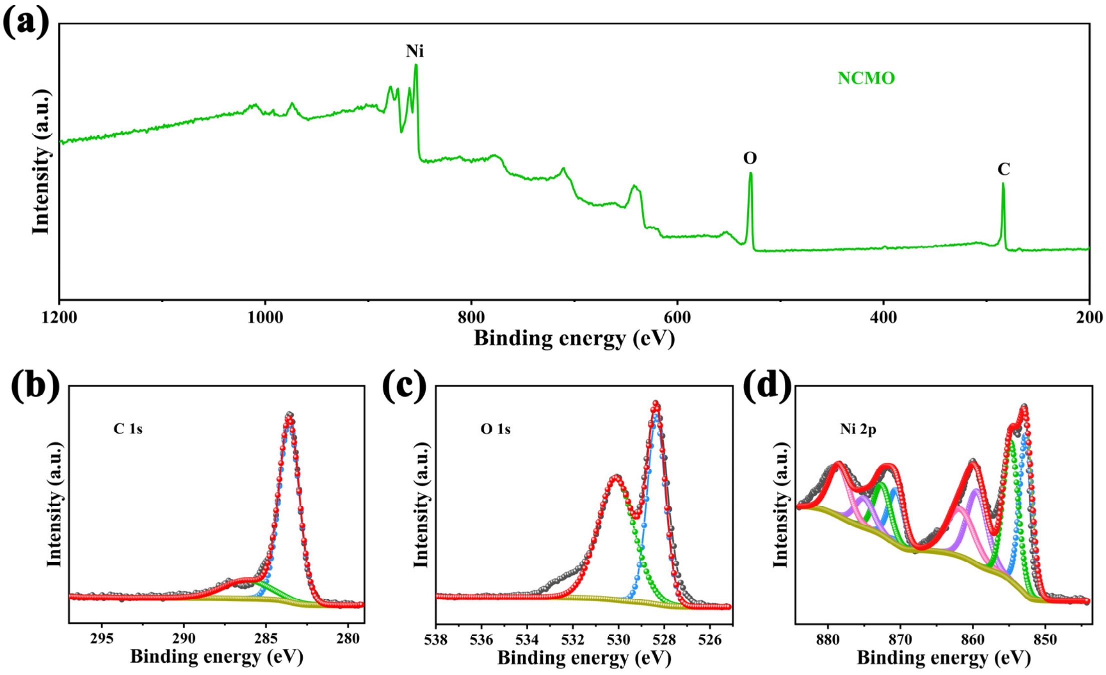

Figure S4. XPS spectrum of NMO

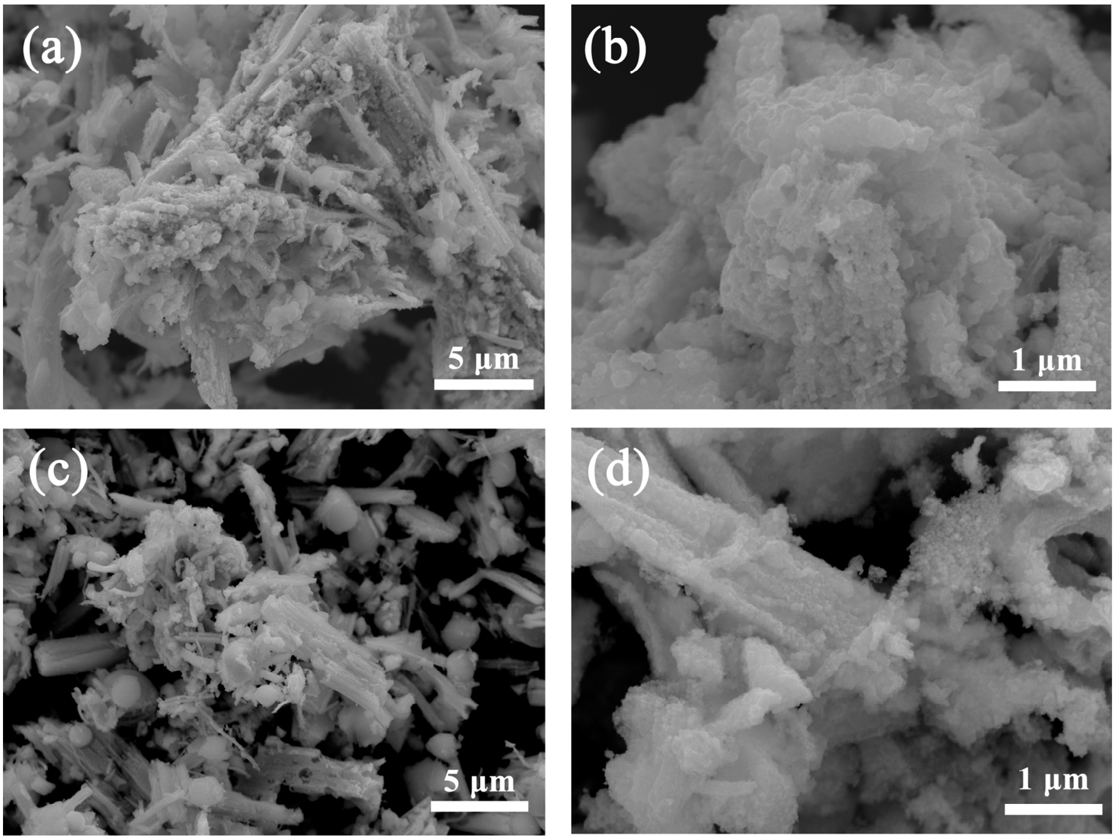

Figure S5. (**a, b**) SEM images of NMO (3°C min^{-1}); (**c, d**) SEM images of NCMO (3 °C min^{-1})

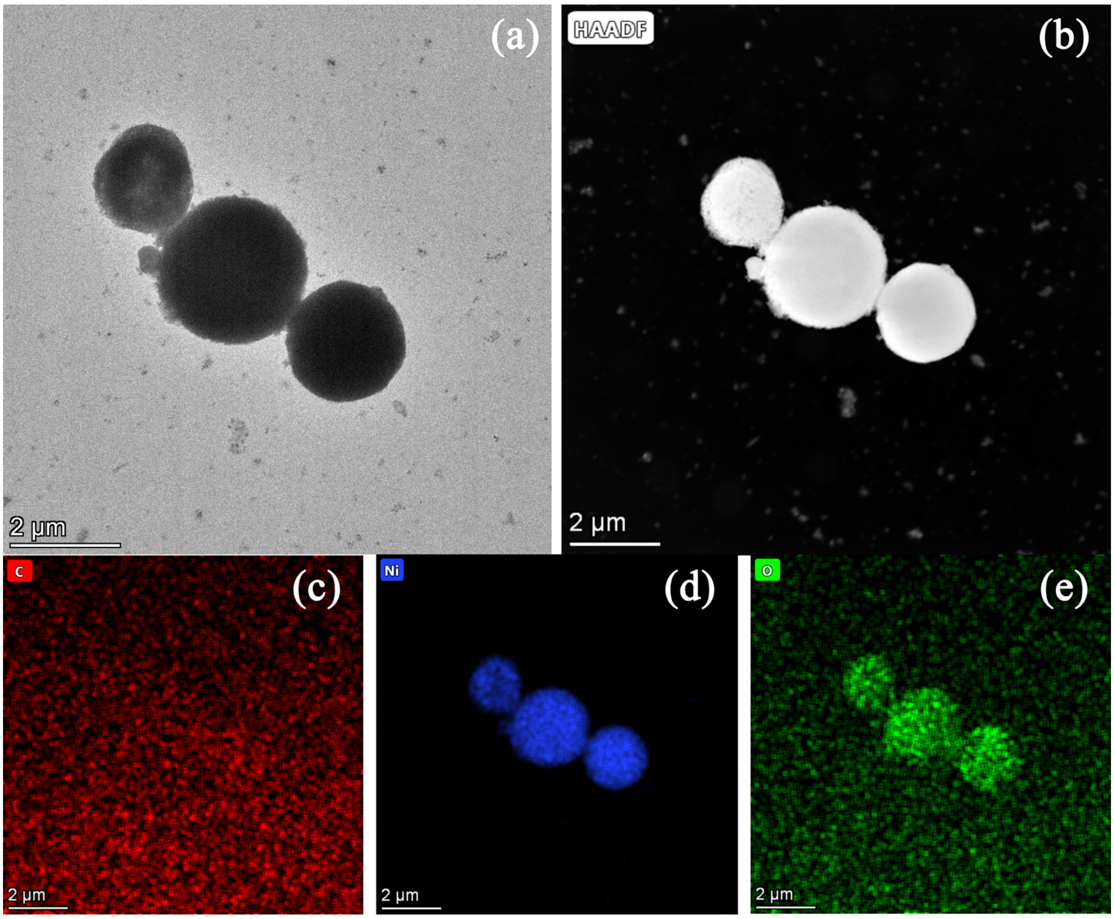

Figure S6. **(a)** TEM image of NMO; **(b)** HAADF-STEM image of NMO; **(c, d and e)** elemental mapping of NMO
